# Experiences of women of childbearing age regarding Implanon provision in South Africa

**DOI:** 10.4102/phcfm.v15i1.3860

**Published:** 2023-07-03

**Authors:** Modiegi D. Motlhokodi, Thifhelimbilu I. Ramavhoya, Mmapheko D. Peu

**Affiliations:** 1 Department of Nursing Science, Faculty of Health Sciences, University of Pretoria, Pretoria, South Africa; 2 Department of Health, Bojanala District, Rustenburg, North-West Province, South Africa; 3 Department of Nursing Science, Faculty of Health Sciences, University of Limpopo, Polokwane, South Africa

**Keywords:** healthcare providers, childbearing, provision, Implanon contraceptive device, healthcare facilities

## Abstract

**Background:**

The World Health Organization has stated that millions of women of childbearing age in developing countries who are not planning to be pregnant are not utilising modern contraceptives such as long-term contraceptives, including Implanon. South Africa had a high rate of women of childbearing age who used Implanon as one of long-term contraception methods from its introduction in 2014. Familiar reasons for women to not use modern contraceptives involved a lack of healthcare facilities, supplies and trained healthcare workers in their area to provide effective contraceptive services in South Africa.

**Aim:**

This study aimed to explore and describe the experiences of women of childbearing age regarding Implanon provision.

**Setting:**

The study was conducted in primary health care facilities of Ramotshere Moiloa subdistrict, South Africa.

**Methods:**

Qualitative, descriptive phenomenological approach was used in this study. Twelve women of childbearing age were purposively sampled. Childbearing age refers to woman in their reproductive ages who will not be regarded as high risk for pregnancy. Semi-structured interviews were utilised to collect data and five Colaizzi’s steps of data analysis were used. Data were collected from 12 of 15 selected women of childbearing age who had experience in utilising Implanon contraceptive device. Data saturation was reached after interviewing 12 participants as the information was coming out, repeatedly.

**Results:**

Three themes with subthemes emerged from the study, namely period of Implanon use, experiences of obtaining information regarding Implanon and healthcare experiences related to Implanon.

**Conclusion:**

It was evident that a lack of effective pre- and post-counselling, eligibility screening and poor management of severe side effects are contributory factors that led to early removal and decline in uptake of the said method. There is also a lack of effective comprehensive Implanon training to some of reproductive service providers.

**Contribution:**

It may increase the number of women who still want to use Implanon as a reliable method.

## Introduction

Effective, affordable and high-quality sexual and reproductive healthcare services including health information on wide ranges of contraception methods are important to cover the rights and well-being of women and girls, men and boys.^1^ As such, the World Health Organization indicated that there are reasons why promoting family planning help future results for women of childbearing age, their families and communities.^1^ One of the identified reasons was the prevention or eradication of pregnancy-related health risks for women and young girls including infant death rate. Bahamondes et al.^2^ supported this statement when they revealed that women in sub-Saharan African countries have many myths and misperceptions related to the utilisation of contraceptives such as affecting fertility if used for longer periods. As a result, these countries are experiencing a high fertility rate, about 4–5 births per woman. In Ethiopia, researchers identified that the husbands of women who were utilising the Implanon contraceptive device wanted more children and consequently opposed the method, which resulted in huge removals of Implanon before its stipulated period of 3 years.

Low utilisation of contraceptives was also recognised in Uganda, Nigeria, Namibia and also in South Africa as it falls in sub-Saharan Africa.^2^ These countries still experience high rate of teenage pregnancies with high risk of maternal deaths and termination of pregnancies (both legal and illegal).^2^ High supply of contraceptives in all country parts, especial rural area, could have prevented such mentioned problems.^2^ Regardless of the availability of contraceptive devices in all public and private healthcare institutions, researchers have identified low uptake of the Implanon device as a contraceptive method. It is against this background that the researchers conducted this study to investigate why there was high-rate removal and low uptake of Implanon before its anticipated 3 years.

Implanon contraceptive device is regarded as an effective, economical, accessible and trustworthy long-acting contraceptive method.^1^ The non-success of Implanon is 0.05% for complete use, so as opposed to other methods of contraception it has a low failure rate.^3^ The National Department of Health Contraception guidelines and policy stated that trained and skilled healthcare providers should provide all women with comprehensive contraceptive services in all healthcare facilities.^3^ Regardless of this statement, women of childbearing age still experience poor provision of Implanon contraceptive device in healthcare facilities. Such poor provision might be influenced by the knowledge and skills of healthcare providers as some were not trained in the insertion and removal of the method. As such, when women seek the reproductive healthcare services, some did not have access as those who were trained were not available.^3^ Regardless of its effectiveness, 15% of women of childbearing age in South Africa are removing it a few months after insertion. Few of the mentioned reasons for removing it were side effects similar to other progesterone-based contraceptives such as irregular bleeding and amenorrhoea.^4^ Some of the women were expected to continue with the method regardless of severe side effects they experienced with no management to stop those side effects.^4^ This brought high rate of early removals of Implanon before its anticipated period and decline in its uptake in 2017 as compared with the year it was introduced in 2014. This attracted the researcher to conduct this study in order to explore the experiences of women of childbearing age in selected healthcare facilities in North West Province, South Africa.

## Research methods and design

### Research design

Qualitative, descriptive phenomenological method was utilised to get information from the participants. The qualitative approach provided the researcher with an opportunity to explore the data that participants revealed, offering greater understanding on how the participants experience the provision of Implanon device.

### Study setting

The study was conducted in Ramotshere Moiloa subdistrict, which is one of the four subdistricts of Ngaka Modiri Molema District in the North West province, South Africa. It was identified as one of the subdistricts with high number removals and low uptake of Implanon device. The subdistrict is situated in the town of Zeerust, comprising 21 primary health care (PHC) clinics of 22 villages. Some PHC clinics operate for 8 h and others for 24 h providing reproductive health services. The healthcare facilities are operated by 4–16 professional nurses, one or two trained in Implanon device, while some facilities do not have Implanon-trained nurses.

### Population

The population of this study comprised women of childbearing age of 18–40 years who were using Implanon contraceptive device and those who removed it earlier before its anticipated 3 years and reside in Ramotshere Moiloa villages. A non-purposive sampling was utilized to select participants. The researcher recruited participants because of their certain knowledge and experience regarding the problem under study. Random selection of 15 participants was performed and it depended on their availability. The researcher conducted interviews with women from four of seven selected clinics that provided Implanon device and other contraceptive as they had high number of early removals and low uptake of Implanon. Data saturation was reached after interviewing 12 participants as the information was coming out, repeatedly.

The researcher conducted interviews with women from four of the seven selected clinics who met inclusion criteria and agreed to participate in the study. The researcher prevented sampling bias by gathering data from women in different clinics.

### Data collection

Semi-structured individual interviews were utilized to collect data from the participants. The clinical staff members briefed the participants about the study, and upon the consent of the participants, the researcher was provided with the contact numbers of the participants. The researcher arranged appointments with participant by phoning them and interviews were held at local clinic of the village where participants were residing. Interview guide with one wide, central question was established as follows: ‘What was your experience regarding provision of the Implanon contraceptive device in the healthcare facility where you received family planning services?’ Translated in Setswana ‘*Ke maitemogelo a feng a o kopaneng le one ga o ne o lefelwa thibela pelegi ya Implanon?*’ The researcher used English and sometimes translated it to Setswana as these two languages are dominating in Ramotshere Moiloa subdistrict. Probing questions followed the responses from the participants and assisted the researcher to gather more information as they were expressing their experiences with the provision of Implanon contraceptive device. An audio recorder was utilised during interviews with permission from the participants in order to capture the information and later transcribe it with the intent to analyse data. The recording of speech by audio tape assisted the researcher in capturing real stories highlighted by participants in case the researcher missed some information when taking field notes. Interview session with each participant lasted for 30–45 min. Data were collected in October and November 2020 for 6 weeks. Interviews were held under strict coronavirus disease 2019 (COVID-19) protocols to prevent spread of infections.

### Data analysis

Data analysis was sensitively executed with the intention of providing structure to and obtaining meaning from the research data. Regarding the qualitative research approach, the researcher utilised the Collaizzi’s five steps method of data analysis as it was regarded as best acceptable for this study.^5^ It allowed new understanding to be divulged and provided intuition into the phenomenon of the research by guarantying credibility and reliability of the study outcome. Regarding phenomenology, data analysis goes concurrently with data collection with the intention to honour and preserve the participants’ lived experiences. The researcher paid more attention to the experiences of women regarding provision of Implanon as phenomenon under investigation.

Collaizzi’s five steps were utilised as indicated: audio recorded interviews were verbatim transcribed and compared with field notes. The researcher listened to the audio recorded interviews carefully to ensure if transcripts are reliable and rectify where applicable. Every transcript was scrutinised and main statements were drawn out utilising coloured pens, thus assisting the researcher to arrange, identify, recuperate and analyse data precisely. Researcher assigned meaning and grouped information in line with their colours, which were combined with gathered information to ensure that every statement was accurately classified. Theme and subthemes that surfaced from the constructed meanings were grouped together to design concerted meanings, which were unambiguous statement of the significant structure of the phenomenon and then submitted to an independent coder. An independent coder also identified the same meanings and clustered them together to categorise familiar experience of the participants.

### Trustworthiness of the study

To ensure the trustworthiness of this study, the researcher utilised measures to promote rigour, which are credibility, dependability, conformability, transferability, bracketing and reflexivity. The researcher ensured credibility by collecting data from diversified samples based on age and experiences and took field notes in addition to the use of the audio tape, which comprised each participant’s individual attributes and environment. Verbatim transcripts were developed to ensure that the data were accurately reflecting the directive statements. In safeguarding dependability, the researcher held interviews with the participants who met inclusion criteria to the point of data saturation. An independent coder was hired to assure the quality of data together with its analysis. Regarding conformability, researcher ensured that it was adhered to by utilising investigation audits where the auditor examined the censorious incidents of the investigation. With transferability, sufficient details about procedures, data collection and analysis tools were provided to make sure that the readers had more information about the conducted study and that the study will be generalised to other similar contexts. This permitted them to evaluate the research impartially and enabled them to direct it to divergent settings. Transferability in this study was ensured during the pilot study, as researcher got similar results and the design together with methodology used in the study were clearly outlined. To recognise bracketing in this study, the researcher is a professional nurse with 7 years’ clinical experience with broad experience of working in multiple clinics with reproductive services. To obtain bracketing, the researcher set aside her preunderstanding and being non-judgemental to have new understanding on data revealed. This has given the researcher an opportunity to conduct the study with a clear mind, which prevented bias. By applying reflexivity for this study, the researcher incorporated and reflected on participants’ responses and decisions made during the interviews by participants to prevent personal bias in making judgements.

### Ethical considerations

Ethical clearance to conduct this study was obtained from the University of Pretoria, Faculty of Health Sciences Research Ethics Committee (no. 595/2020).

## Results

### Biographical profile of participants

Data were collected from 12 of 15 selected women of childbearing age who had experience in utilising Implanon contraceptive device. The participants were aged between 18 and 40 years. They were black African women staying in villages found in Ramotshere Moiloa subdistrict. All of the participants were single and their level of education ranged from the secondary to the tertiary levels. Table 1 presents the themes and subthemes that emerged from the study.

**TABLE 1:** Themes and subthemes in the use of Implanon contraceptive by women.

Themes	Subthemes
1. Period of Implanon use	1.1. Implanon usage for three anticipated years 1.2. Implanon removed in less than 3 years because of experienced side effects
2. Experiences of obtaining information regarding Implanon	2.1. Information obtained from healthcare services (clinics) 2.2. Information obtained from others
3. Healthcare experiences related to Implanon	3.1. Effective versus ineffective healthcare during insertion and removal of Implanon 3.2. Insufficient management of side effects

### Theme 1: Period of Implanon use

This study revealed that nine women used Implanon contraceptive device for a duration of 3 years to lessen frequent visits to the primary health care facilities. From the same study, most women discontinued the device early because of intolerable side effects. From this theme, two subthemes emerged as indicated in the next section.

#### Subtheme 1.1: Implanon usage for three anticipated years

The Implanon is a long-term contraceptive and many women of childbearing age utilise it as it is safe and affordable. The participants revealed that they have utilised the method for 3 years, removed it and inserted new one as they did not experience any side effects. One participant said:

‘I inserted Implanon on 31 July 2017 and removed it on 05 August 2020 because three years was expired. I inserted it again on 19 September 2020. It is taking me well I do not get dizziness and it is good to me.’ (Participant 1, 37-year-old, single)

Another participant said:

‘I used it for three years full, inserted it on February 2014 and removed it on February 2017. I had no problems with Implanon during those three years, no side effects. I was eating well, no weight gain.’ (Participant 9, 36-year-old, single)

Participant 3 agreed and said that she utilised a short-term method first and then moved to Implanon as a long-term method of contraception. Since she started utilising the method, she had not experienced any side effects. She said:

‘I used Nur-Isterate before then I decided to use Implanon to avoid coming to clinic frequently and it is for three years. As it is, I do not experience any problem with it. It is the best method to use.’ (Participant 3, 28-year-old, single)

Participants in this study have used Implanon contraceptive device for a duration of 3 years and more. The women indicated that they went back to the facilities for the renewal of the new Implanon contraceptive device after the expiry of the first insertion.

#### Subtheme 1.2: Implanon removed in less than 3 years because of experiencing side effects

Study participants revealed that they had removed the method in less than the 3 anticipated years as they experienced side effects. Some reported that they had used it for 6 months, and others removed it the same year it was inserted because of the reported side effects. They said:

‘I used it for six months and I started to have side effects, menstruating too much to explain it, I will be menstruating twice in a month, then I started to experience dizziness I became sicker frequently and gaining weight.’ (Participant 2, 31-year-old, single)

‘I removed it same year 2015, in less than a month I had continuous menstruation and not stopping. It was just that when you bleed too much you get dizziness.’ (Participant 7, 24-year-old, single)

Other participants concurred by saying that they experienced skin changes and weight gain and did not have their monthly periods, which led to early removal of the Implanon. They said:

‘I inserted it on the 16 September 2014 and removed it on 12 October 2015. I had general body pains, lower abdominal pains, not menstruating. I started to gain weight and my skin colour changed from being coffee colour to be black. *Ke ne ke lapa thata* [*I felt severe tiredness*] that usually comes when I was about to menstruate as a sign that I could be menstruating, but no bleeding came out.’ (Participant 8, 39-year-old, single)

‘I used Implanon for seven months only. I was not menstruating, gained weight, my skin become darker, and I am naturally very light skinned colour.’ (Participant 10, 27-year-old, single)

In summary, some participants used Implanon for less than 3 years and removed it as a result of side effects.

### Theme 2: Experiences of obtaining information regarding Implanon

Healthcare providers are front liners in the healthcare facilities and have an impact on contraception uptake by women. Healthcare providers and healthcare facilities are designated areas where women of childbearing age should obtain information about their reproductive health, including information on Implanon. From this theme, two subthemes emerged as indicated below.

#### Subtheme 2.1: Information obtained from healthcare facilities (clinics)

Contraceptive education is a very significant tool of comprehensive healthcare to prevent unplanned pregnancy, the high rate of safe and unsafe termination of pregnancy and maternal death. Most of the women in this study reported that they had obtained information regarding Implanon as a method of family planning during the workshops they attended. Other participants reported that they had heard about the method when they brought their children to the clinic for well-baby clinic. This information was supported in the following quotations:

‘I heard about Implanon when attending workshop, its importance and it takes three years without frequent clinic visits like to come for injection or monthly contraceptive pills.’ (Participant 4, 32-year-old, single)

‘I heard about Implanon in the clinic when I brought my second child, they said it is safe and that encouraged me to use it as method of preventing pregnancy. It last for three years because I became pregnant when I was using pills and made some mistakes.’ (Participant 6, 37-year-old, single)

Another participant added that she was informed about the side effects of the method, hence she inserted it understanding what to expect. She said:

‘I heard about Implanon in 2014 at Moshana clinic when it was presented. They explained its side effects. They told me everything about it even during in-service training of staff because I was employed at the clinic. They told me everything about it and I inserted it expecting those side effects.’ (Participant 12, 34-year-old, single)

In this study, participants indicated that they got the information about Implanon from healthcare settings. As such, the information assisted them on the suitable method of contraception to choose, thus preventing unwanted pregnancy.

#### Subtheme 2.2: Information obtained from others

There are many factors affecting women’s use of contraceptives, such as insufficient information. This influences their choice regarding contraceptive use. There are risks such as unintended pregnancies. In this study, some participants reported that they had obtained information on Implanon as a method of family planning and its side effects from their friends. Other participants reported that they have heard from other patients when they were discussing Implanon and its side effects such as vomiting and dizziness. Regardless of their experiences, the woman decided to use the method and they did not experience any of those side effects:

‘I heard about the method from her friends, they referred to the method as used for three years and that it is not taking them well, they were vomiting and having dizziness and I decided to try it and didn’t have side effects.’ (Participant 3, 28-year-old, single)

Another participant alluded that she heard the information on Implanon being discussed where she stays. She also highlighted some of the side effects such as weight gain:

‘I heard people in the village talking about it and the other one said it made her to gain weight because she had had low weight for her age. I wanted to gain weight because people would start to think that I am sick and so forth.’ (Participant 6, 37-year-old, single)

In this study, participants indicated that they have obtained information about Implanon contraceptive device and its side effects from other people, for example, their friends. They decided to use it because of its long-term advantage.

### Theme 3: Healthcare experiences related to Implanon

Participants reported that they had received proper care during insertion and removal of the Implant. However, some participants indicated insufficient management of side effects and education regarding how the method works and what to expect when using the method. From this theme, three subthemes emerged as alluded below.

#### Subtheme 3.1: Effective versus ineffective healthcare during insertion and removal of Implanon

Participants reported mixed feelings regarding the insertion and removal of the implants. Some participants indicated that nurses at the clinic had difficulties in removing the implants as it had been inserted deeply, although some indicated that it was not difficult for nurses to insert and remove the implant. Briefly, most of participants had experienced a good insertion and removal of Implanon.

They said:

‘I did not experience any problem regarding Implanon insertion and removal. At the clinic they injected me not to feel pain on insertion and removal.’ (Participant 3, 28-year-old, single)

‘During removal I was injected not to feel pain but, sister struggled to remove it as it hidden too deep in my skin and removal took long time.’ (Participant 4, 32-year-old, single)

Another participant added that she felt pain when the Implanon was removed:

‘I was injected not to feel pain and sister told me that I am bleeding too much and as the time went on it became painful until she found and remove it. Even after removal, I was still feeling sensitiveness on the scar where it was inserted.’ (Participant 11, 29-year-old, single)

In this study some of the participants were satisfied by the way the insertion and removal of Implanon was handled, although some experienced pain on removal.

#### Subtheme 3.2: Insufficient management of side effects

The participants of this study indicated that they always reported the side effects of Implanon they were experiencing. Some mentioned that they were advised to remove it and others were given treatment that did not assist them. They indicated that nurses told them during insertion to return to the clinic for removal should they encounter problems. The women said:

‘Hmm … at the clinic, the nurse who inserted the implant told me that, if I experience any problems such as prolonged bleeding, I could come to remove it so I went back to the clinic for nurses to remove it.’ (Participant 5, 24-year-old, single)

‘I went back to the clinic because I saw that I was losing weight. So, at the clinic, nurses did not give me any problem to remove it because I told them I am losing too much weight.’ (Participant 6, 37-year-ol, single)

Another participant indicated that she was not bleeding heavily but the fact that she would find her underwear stained without expecting blood made her uncomfortable:

‘I reported to nurses at the clinic that I’m bleeding almost every day, though sometimes it will stop for two days, I will find my underwear stained with blood the next day. I felt uncomfortable about it and life was no longer easy for me. But since I was not bleeding heavily, I think if they could have given me more information and treatment to stop bleeding, I would not have removed it.’ (Participant 7, 24-year-old, single)

On the contrary, another participant indicated that nurses reassured her that with time, the side effects would fade away, but she could not wait for another month and decided to remove the implant:

‘Yes, and they told me maybe after a month it will be better … I could not wait for another month. I asked them to remove it that day that I went to the clinic.’ (Participant 12, 34-year-old, single)

This study shows that the side effects experienced by most women were not managed accordingly, which led them to remove their Implanon before its 3 anticipated years.

## Discussion

### Period of Implanon use

Women of childbearing age who participated in this study have shown understanding that Implanon contraceptive device can be used for a period of 3 years. Some of these women did not experience side effects, while others indicated that they had difficult time with Implanon side effects while utilising it. Participants who did not experience side effects continued to use it for its anticipated 3 years and reinserted it when it was time for its replacement. They recommended it as a suitable method as it is a long-term pregnancy prevention contraceptive. Espey et al.^4^ concurred with the findings that the most effective methods of reversible contraception are the so-called long-acting reversible contraceptives, intrauterine devices and implants. Another study conducted by Pillay et al.^6^ revealed that in general women’s experiences with the Implant were rated best by 74% of those who continued to utilise it, most of whom indicated that they did not experience any side effects. Some of the participants mentioned that they utilised Implanon for less than 3 years. Their reason was that they had not known about the troublesome side effects that forced them to remove it before its anticipated 3 years. The mean period of Implanon utilisation was 1 month to 2 years.

### Experience of obtaining information regarding Implanon

The findings of the study revealed that participants obtained information about Implanon from healthcare providers and were convinced to utilise it, as it was regarded to be the best long-term effective method. Contrary to the results of this study, the National Department of Health indicated there are critical missed opportunities for information provision and education on reproduction that can be provided to women in different places of health and educational settings.^3^ This influences them to decide about contraceptive methods use and their risks. Other participants heard about Implanon from their friends. Their friends told them how it is inserted along with its side effects. Regardless of the information they obtain about Implanon, the participants made decision to utilise the method to prevent pregnancy. Furthermore, most of the participants in this study heard about Implanon from healthcare providers. Moodley et al.^7^ conducted a study on the prevalence and predictors of Implanon uptake in Ugu North subdistrict in the KwaZulu-Natal Province. Their study concurred with the findings of this study where 86.7% of participants, were aware of Implanon contraceptive device as a contraceptive modality with more than 57.3% of patients obtaining information about it from healthcare workers.^7^ Provision of information by healthcare providers and friends assisted women to make a better choice of using Implanon, which prevented unwanted pregnancy.^3^

### Healthcare experiences related to Implanon

This study revealed that majority of nurses are skilled in inserting and removing Implanon contraceptive devices. This was indicated by most participants that Implanon insertion and removal was very good as they did not experience problems with such services. It was concluded that training in procedures and compliance with procedural instructions are important in reducing risks, including deep insertion and damage to neurovascular structures.^8^ Only two women in this study indicated a bad experience during the removal of the Implanon device that it was too deeply inserted and nurses struggled to remove it. Pillay et al.^6^ concurred with this finding and stated that women reported ongoing pain on the insertion site, rashes and swelling, which further led to the removal of the Implanon.

In this study, participants complained about the side effects to the nurses in the clinic but they were not managed accordingly. They further mentioned that the nurses were advising them to remove the Implanon device instead of treating the side effects. From the findings, it was evident that participants had received inadequate health education regarding the side effects of Implanon. Such education was anticipated prior to the insertion of the Implanon contraceptive device so that women had an understanding of how it works. In alignment with this study findings, Mkansi^9^ found that secondary school-going girls at Bokamoso School in Polokwane District had insufficient knowledge about Implanon, how it works and its side effects.^10^ Rabopape et al.^11^ revealed similar findings to this study, which showed that a lack of knowledge about Implanon’s side effects, hence contributed to low uptake by adolescent girls in the Limpopo province.^9^ Adeagbo et al.^12^ concurred with this study finding, as women required detailed and evidence-based counselling that encompasses the likely side effects and other features of the method. Mghobozi^13^ also supported this study finding that side effects management given to women was for the shorter period and indicated that there should be long-lasting treatment to tackle those side effects to promote the uptake of Implanon device for 3 anticipated years, hence prevention of unwanted pregnancy.^11^

### Study limitations

This study was conducted in a specific situation in a single subdistrict that narrowed the scope in the research. Twelve participants made up a sample of the study and they were from five healthcare facilities in the subdistrict. The researcher struggled with getting contact numbers of eligible participants in one of healthcare facilities, as they were not recorded in registers and those participants could not be interviewed. The research was conducted in public healthcare facilities and findings could not be stretched out to private healthcare facilities where Implanon contraceptive devices are also provided. The COVID-19 pandemic contributed to some of the selected participants not attending interviews as they were afraid of being infected by the virus.

### Recommendations

Training of healthcare providers regarding Implanon contraceptive device must be intensified. Policies and standard operating procedures must be implemented effectively and reviewed frequently to beef up skills of healthcare providers. The National Department of Health should collaborate with cellphone network providers and the media, such as television and radio, to educate the society about the advantages of utilising Implanon contraceptive device. Information about Implanon contraceptive device should be included in maternity case records for women to obtain information and decide if they would use it after giving birth. Experimental studies on the content of Implanon could be carried out to modify them so that side effects could be reduced to accommodate all women of child-bearing age including those who are on chronic treatment. More researches are recommended regarding Implanon contraceptive device.

## Conclusion

There should be effective management of possible side effects of Implanon and provision of proper information including counselling about Implanon contraceptive device. The findings of the study revealed that side effects and a lack of information are major contributing factors to low utilisation and early removal of the method before its anticipated 3 years. As alluded to by participants in this study, difficulties in the insertion and removal of Implanon by other healthcare providers were noticed as poor provision of Implanon. As such, from their experience, women of childbearing age were able to relate their stories about Implanon contraceptive device as an effective method of preventing pregnancy for 3 years.
